# Examining the Effectiveness of a Web-Based Intervention for Depressive Symptoms in Female Adolescents: Applying Social Cognitive Theory

**Published:** 2019-08-19

**Authors:** Babak Moeini, Saeed Bashirian, Ali Reza Soltanian, Ali Ghaleiha, Malihe Taheri

**Affiliations:** ^1^Social Determinants of Health Research Center, Hamadan University of Medical Sciences, Hamadan, Iran .; ^2^Department of Public Health, School of Public Health, Hamadan University of Medical Sciences, Hamadan, Iran; ^3^Modeling of Noncommunicable Diseases Research Center, Hamadan University of Medical Sciences, Hamadan, Iran; ^4^Department of Biostatistics, School of Public Health, Hamadan University of Medical Sciences, Hamadan, Iran; ^5^Research Center for Behavioral Disorders and Substances Abuse, Hamadan University of Medical Sciences, Hamadan, Iran.

**Keywords:** Depression, Social cognitive theory, Web-based intervention, Adolescents

## Abstract

**Background:** Depression is a serious mental health illness among adolescents especially girls. Web-based treatment can possibly become a solution for reducing mental health problems in adolescents. This study is one of the first trials aimed to evaluate the efficiency of web-based depression improvement program among female adolescents based on the Social Cognitive Theory (SCT).

**Study design:** Randomized Controlled Trial.

**Methods:** A six-month randomized controlled trial based on the SCT was implemented in female schools in Hamadan City, west of Iran in 2018 on 128 female students with mild to moderate depressive symptoms (CES-D =10-45). They were randomly assigned to either intervention or control group (n= 64 in each group). Depression improvement program was implemented through the website via short videos, animations and Power-Point slides. Depression was evaluated using Center for Epidemiologic Depression Scale. A researcher-made questionnaire based on the sheerer and Perceived-Social-Support-Scale-Revised (PSSS-R) questionnaires were used to evaluate the SCT constructs. The data were analyzed using SPSS software.

**Results:** The intervention program resulted in the improvement of depression in 12 wk based on Intent-to-treat analyses. Nevertheless, these achievements seem to have decreased by 24 wk in intervention group. The intervention increased the mean scores of the constructs of social support and self-regulation from baseline to 12 wk in the intervention group (*P*<0.05). The intervention had no effect on outcome expectations and self-efficacy. There were no statistically significant associated between theory constructs changes and changes in depression (*P*>0.05).

**Conclusion:** The web-based intervention improved depression in female students. Future training using strategies for the sustainable improvement of depression in female students are needed.

## Introduction

Depressive disorders are main health problems^1^ and are the most common psychological disorders in adolescents with a prevalence rate of 13%-43.5%^2^. Girls have higher levels of depressive symptoms than boys^3^. Mild and moderate depression is also ordinary, with a prevalence rate of 36.2%-40.9% among Iranian adolescents^4^.

Development of preventive interventions based on suggestion of WHO can decrease the burden of depression, because of the high prevalence rate, serious consequences and economic burden of depression^5^. Adolescents encounter various obstacles to find professional help. The prolonged waiting lists, limited number of mental health professionals, direct and indirect costs of treatment, low mental health literacy and fear of stigma are some barriers to seeking help for mental illnesses^6^. As a solution, the internet-based interventions can assistance with achieving untreated target groups. Significantly, due to the growing accessibility to the internet, it can remove barriers to treatment for depressed individuals and healthcare professionals^7^. Studies on treatment using the Internet for individuals with depression revealed positive outcomes^8-10^. However, there is a limited evidence regarding their effectiveness among Iranian adolescents. To the best of our knowledge, only one randomized controlled trial using new technologies and with mobile application has been conducted on depression prevention in Iran^11^. Of course, web-based interventions have been done limitedly for other adolescents' health issues in Iran. For example, a web-based educational program was conducted to prevention of tobacco smoking among male adolescents^12^.

Web-based interventions with a theoretical framework are more effective^13^. The Social Cognitive Theory (SCT) would be appropriate for the development of a web-based behavior-change interventions^14^.

We aimed to examine the effectiveness of a web-based intervention for depressive symptoms in female adolescents; Application of the SCT that to our knowledge is one of the first studies of this type. Significant reduction in female student’s depression (mild and moderate level) and enhancements in SCT constructs from baseline to 24 wk will be found and changes in SCT constructs would be positively associated with changes in depression levels.

## Methods

### Participants

This was a randomized controlled trial on female high schools in Hamadan City, west of Iran in 2018. The prevalence of mild depression was reported 22%^4^. Therefore, the sample size was estimated 64 participants in each group given a confidence interval of 95%, power of 90% and attrition rate of 20%. The simple random sampling method used for sampling.

This report is part of a major study of factors associated with depression. In the first phase of the study that implemented in Hamadan in 2017, female high schools with high prevalence of depression identified^15^. To attain the sampling purpose of present study, among four schools with the highest rates of depression, two schools were randomly allocated to the intervention group and two schools were allocated to the control group. Students with mild to moderate depression level in selected schools were listed according to their student codes. Of which 128 students were randomly selected and were assigned into the groups (n=64 students in each group).

The selected students were informed regarding the study aim and method. Their parent/guardian were asked to sign the informed consent form. This study was approved by the Ethics Committee of Hamadan University of Medical Sciences (IR.UMSHA.REC.1394.548). The trial was also registered under the code of IRCT2016110427488N1.

**Figure 1:**
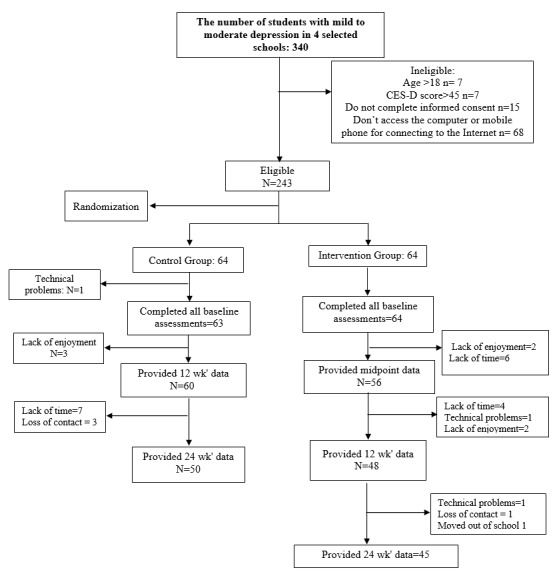
Flow diagram of participant recruitment and retention

Inclusion criteria were: female student, age 15-18 years, having access to Internet and the Center for Epidemiologic Studies Depression Scale (CES-D) score between 10 and 45 (mild and moderate depression). Individuals with major depression were excluded from the study and were recommended to contact a psychiatrist. Other exclusion criteria were: do not access the computer or mobile phone for connecting to the internet, taking antidepressants, current participation in an intervention targeting depression; and moving to another school. Figure 1 displays the process of sampling and recruitment.

### Intervention

This study was performed to examine the effectiveness of the DAD (Dorehye Amozeshie Dokhtaran) course. This web-based education program had two-phases. In the first phase, qualitative study (2 interviews and a focused group discussion) was conducted on 45 female students and their needs of depression improvement website applications were identified. In the second phase, based on the data collected in the first phase, a preliminary website was development. Table 1 shows the themes extracted from the qualitative study and the included features or applications on the educational web site to address these themes.

**Table 1:** Themes developed from the qualitative phase used for website development

**Theme**	**Sample quotes**	**Website application**
Professional support	If I can use the comments of a psychiatrist throughout the program, it's would be much better	Online chat opportunities privately and publicly with a psychiatrist
Active engagement	I like to do some assignments, so I can do and send them and receive feedback	Make it possible to upload assignments after each session and send personal feedback to participants
Modeling	If I see similar students of people like myself who express their positive experiences to improve depression, I would love it.	video of a successful girl student who dominates her depression and expresses her strategies and experiences
Meditation exercise instruction	I heard that meditation and yoga helped people with depression. It is very good if you can have videos about it	Meditation exercise videos
More information	In addition to seeing the video and the website, I would like to have access to the main sources such as books	Ability to upload pdf books in "more resource "section
Awareness raising	I would like to get some information about depression. My information on this area is little and this can affect my confidence.	Videos in simple words to raise awareness such as videos of university professors’ speeches Self-efficacy applications
Depression improvement self-regulation applications	Goals such as “where you are today” or “this is your target goal for today.” Or if you don’t log in for a couple of days, something like, “well, since you missed your target day, this is your target now.”	Goal setting applications

### DAD program

“Dorehye Amozeshie Dokhtaran” was provided in “Electronic Learning Management System” section of Hamadan University of Medical Sciences’ web site (http://lms.umsha.ac.ir/AttachmentView/24/326/3/326/Urlnull). This program was based on cognitive-behavior therapy methods to handle mild and moderate depression. For each participant, a private username and password were defined so that they could access the educational website privately.

The DAD internet intervention program was grounded in the SCT constructs as a behavioral health theory that explains behaviors in terms of a triadic and reciprocal model, in which an individual’s behavior, personal factors and the environment interact and influence each other^14^. The DAD program contains seven core modules including introduction and assessment, awareness-raising, positive psychology, problem-solving, thoughts and feelings, relaxation, physical exercise and lifestyle modifications. Each module was planned in a multimedia format that includes videos, real-life examples, pictures, animations, and assignments. The participants were encouraged to identify their own unproductive, insubstantial beliefs and were trained to convert these into reasonable and beneficial thinking. Doing enjoyable daily actions was also encouraged, and a mood assessment form was filled out daily for understanding the relationship between the mood level and pleasant activities. The students also had access to supplementary resources about the effects of proper nutrition on depression, assertiveness skills, health anxiety, and sleep improvements. Table 2 shows designed applications associated with the specific constructs of the SCT.

**Table 2:** Applications of the Social Cognitive Theory to intervention modules

**Social cognitive theory construct**	**Modules related to the web-based program**
Self- efficacy	● A video about a female student who managed to overcome her depression and transfer positive experiences (Modeling) ● Video about sub skill demonstration, instruction, and ● enactment with feedback for an enjoyable activity (physical activity) ● Video interviews to raise awareness about depression ● Videos about more practice of challenging thoughts
Outcome expectations	● An animation about the benefits and effectiveness of treating depression and the positive impact of overcoming this psychological problem.
Social support	● Online video chats with a psychiatrist, ● Emotional support was provided by other participants and project executives through the telegram group formed for this purpose ● Access to more resources for improving depression in educational website. ● Identifying automatic thoughts and core beliefs video.
Goal-Setting	● Identifying goals for changing activity videos. ● Identifying hierarchal steps to achieve behavioral goals videos. ● Possibility to download “positive thoughts” list by participants at the end of each week. Completing and uploading them and receiving feedback. ● Relaxation (meditation) exercise videos and goal setting to do it 10 minutes every day.

Individuals received gradual admission to the modules and had constant online assistance from a psychiatrist using a secure online messaging system. It consisted of eight 30-min sessions. Homework assignments were given at the end of each session. Participants were directed to complete two sessions per week.

Text messages reminders were sent to the participants via Telegram^®^, the most popular social network in Iran, one-half hour before each session. The participants could share experiences, actively ask questions; freely respond to others in the group.

All participants received free-of-charge online access to the internet-delivery program and no financial imposed on them. The password code automatically expired after six months.

### Measures

Depression and SCT-related structures were evaluated through self-administered questionnaires at baseline, 12 wk and 24 wk after the intervention. The students acquired reminder messages via the telegram group to complete the online questionnaires.

### Depressive symptoms

The CES-D evaluates the occurrence of 20 depressive symptoms in the past 7 d on a four-point Likert scale. The total score in this questionnaire was between 0 and 60, and higher scores represented the higher levels of depression. The online version of the CES-D has been revealed to be a reliable and valid screening tool ^16,17^. The validity and the reliability of Farsi version of this instrument were examined. Cronbach’s alpha coefficient in our study was 0.90^18^.

### Psychosocial Measures

**Social support:**The Farsi version of the Perceived Social Support Scale-Revised (PSSS-R) was used. It was initially introduced^17^and included 12 items on a 5-point Likert scale. Derived sum scores were employed for addressing perceived support from family, friends, and significant others with a score range of 4-20. Higher scores demonstrate high-perceived social support. Reliability and validity of this scale were evaluated^19^. Reliability of this section of SCT scale had estimates for present study (Cronbach’s alpha=0.79).

**Self-efficacy:**The Farsi version of the Sherer's general self-efficacy questionnaire was used. Reliability and validity of this scale were evaluated and reported a Cronbach’s alpha coefficient of 0.83^20^. This 12 items questionnaire asked the participants to indicate their agreement with each item on a five-point scale. Reliability of this section of SCT scale had estimates for present study (Cronbach’s alpha=0.79).

**Outcome expectations:** The Outcome Expectation Scale for depression contained 7-items that requested the participants to rate their agreement with phrases regarding depression (e.g., I feel insufficiency if I look for depression treatment) on a 5-option Likert scale from, 1 (very disagree) to 5 (very agree). Reliability of this researcher made section of the SCT scale had estimated in the present study (Cronbach’s alpha=0.79). The content validity of the tool was confirmed by 5 panel’s experts.

**Self-regulation:**The self-regulation Scale for depression contained 7-items that requested participants to rate their agreement with phrases regarding depression (e.g., I feel worthless if I look for treatment of depression). Items were selected from a study of the self-regulation model of depression^21^. Reliability of this researcher made section of the SCT scale was estimated for present study (Cronbach’s alpha=0.79). The content validity of the tool was confirmed by 5 panel’s experts.

### Website Satisfaction and self-reported website usage 

The participants accomplished a satisfaction survey questionnaire at the end of the intervention; that assessed the students' general satisfaction with the website. This assessment included various topics such as the frequency of using the website, usefulness of the website, preferences of the website and the website overall satisfaction.

### Statistical analysis 

Based on Kolmogorov-Smirnov test, all of the variables met assumptions for normality. Changes in the depression and SCT constructs from baseline to 24 wk were evaluated by using repeated measures ANOVA test. Significant differences between the mean scores of depressions and SCT structures in both groups were analyzed using ANCOVA test. Multiple linear regression analysis was applied to assess the association between variations in the SCT constructs (as independent variables) and participant’s depression scores (dependent variables).

Complete case analyses and intention-to-treat (ITT) analyses were performed. ITT analyses measured data from all participants with available baseline data. Complete case analyses were used for data collected from those participants who completed the intervention (baseline, 12 wk, and 24wk). Statistical significance was set at P<0.05. The SPSS ver. 16.0 (Chicago, IL, USA) was used for data analysis.

## Results

### Baseline characteristics 

Overall, 243 students met the inclusion criteria of which 128 were recruited. The mean age in the intervention and control groups were 16.2±0.68 and 16.5±0.60 yr, respectively. The participants’ baseline demographic characteristics were illustrated in Table 3.

Follow-up data were completed by 45 out of 64 students in the intervention group (29.6% attrition) and 50 out of 64 in the control group (21.8% attrition) at the end of the 24 wk and were regarded as study completers due to providing data at all evaluation periods. Reasons for attrition were: lack of time (n=15), technical problems (n=3), unwillingness with the study (n=7), loss of contact (n=7) and quitting from the participating school (n=1).

### Qualitative findings

The Mean±SD age of female adolescents was 16.7 ± 1.86 years. The conventional content analysis resulted in identifying 276 primary codes, 14 categories, and 7 themes. Themes were as “Professional support”, “Active engagement”, “Modeling”, “Meditation Exercise Instruction”, “More information”, “Depression improvement Goal Setting Applications” and “Awareness raising” (Table 1).

**Table 3:** Demographic characteristics based on study groups

**Variables**	**Control**		**Intervention**		***P*** **value**
	**Number**	**Percent**	**Number**	**Percent**	
Mother education					0.322
>Diploma	4	6.25	3	4.60	
<Diploma	60	93.75	61	95.30	
Father education					0.683
>Diploma	7	10.93	9	14.06	
<Diploma	57	89.06	61	85.90	
Father’s occupation					0.530
Employee	62	96.87	59	92.18	
Unemployed	2	3.12	5	5.81	
Mother’s occupation					0.921
Employee	21	32.81	25	39.06	
Housewife	43	67.18	39	60.93	
School grade					0.732
10	11	17.18	12	18.75	
11	21	23.81	20	31.25	
12	15	23.43	16	25.00	
Pre-University	17	25.56	16	25.00	
Family income $					0.381
<190	43	67.18	44	68.75	
>190	21	32.81	20	31.55	
Field of study					0.990
Mathematic	8	12.50	10	15.16	
Experimental Sciences	13	20.31	11	17.18	
Humanities	11	17.18	11	17.18	
Technical	15	23.43	17	26.50	
Kar va Danesh a	17	26.56	15	23.40	
Place of residence					0.982
Downtown areas	32	50.00	34	53.12	
Suburbs	32	50.00	30	46.80	
living with parents					0.751
With two parents	57	89.06	54	84.30	
With one of the parents	7	10.93	10	15.60	

^a^ A new major in Iranian high schools

### Social Cognitive Theory constructs changes

In the intervention group, the intent-to-treat analyses revealed statistically significant increase in self-regulation and social support from baseline to 24 wk (*P*<0.05). Similar improvements in these variables were found from baseline to 12 wk (*P*<0.05) that statistically significant compared with the control group. No changes in other SCT variables were observed throughout the study (Table 4). Complete case analyses demonstrated the similar result.

### Changes in depressive symptoms 

The intent-to-treat analyses showed that the intervention group reported a statistically significant improvement on the CES-D score at the baseline (Mean=22.6, SD=10.9) to 12 wk (Mean=18.5, SD=14.0). The ANCOVA test showed that the mean score of differences of depression between the groups was statistically significant (*P*<0.05). Nevertheless, these results seem to have attenuated by 24 wk (Mean=19.5, SD=10.9). No changes were found in the control group during this study (Table 4).

**Table 4:** Changes in depression and SCT constructs before and after intervention

**Variables**	**Before intervention**		**Baseline to 12 wk**		**Baseline to 24 wk**		***P-*** **value**
	**Mean**	**SD**	**Mean**	**SD**	**Mean**	**SD**	
Depression							
Intent-to treat	24.6	11.7	18.5	14.0	19.5	10.9	0.031
Complete case	22.1	10.0	18.0	16.0	20.0	11.3	0.310
Control group	22.3	11.8	21.4	15.6	22.5	12.1	0.251
*P* value	0.153		0.004		0.062		
Self-efficacy							
Intent-to treat	2.77	0.95	2.75	1.10	2.76	1.12	0.123
Complete case	2.57	0.84	2.51	0.89	2.55	0.55	0.153
Control group	2.54	0.85	2.51	0.83	2.52	0.84	0.180
*P* value	0.237		0.301		0.371		
Goal setting							
Intent-to treat	2.20	0.61	2.55	0.68	2.42	0.61	0.030
Complete case	2.27	0/35	2.88	0.67	2.52	0.60	0.031
Control group	2.35	0.37	2.40	0.58	2.38	0.61	0.411
*P* value	0.420		0.021		0.023		
Outcome expectation							
Intent-to treat	4.17	0.63	4.15	0.59	4.13	0.59	0.421
Complete case	4.40	0.54	4.30	0.55	4.32	0.56	0.450
Control group	4.42	0.46	4.41	0.35	4.40	0.45	0.411
*P* value	0.159		0.240		0.159		
Social support							
Intent-to treat	2.67	0.95	2.99	1.04	2.95	0.88	0.002
Complete case	2.57	0.78	2.88	1.07	2.88	1.03	0.010
Control group	2.17	0.87	2.19	0.85	2.18	0.88	0.121
*P* value	0.120		0.011		0.071		

### Associations between changes in the depression and SCT constructs

There was no statistically significant association between changes in the SCT structures and depression in the intervention group in terms of self-efficacy (*P*=0.54), goal-setting (*P*=0.1), outcome (*P*=0.83) and social support (*P*= 0.23) (Table 5).

**Table 5:** Multiple linear regression among social cognitive theory constructs and depression

**Variables**	**Coefficient (95% CI)**	***P*** **-value**
Social support	0.11 (0.01, 0.22)	0.231
Self-efficacy	4.83 (2.61, 7.05)	0.542
Outcome expectations	-0.22 (-2.05, 1.61)	0.832
Goal setting	5.18 (0.39, 9.96)	0.101

## Discussion

After a six-month web-based intervention based on SCT, depression decreased among the DAD group after 12 weeks. The designed modules in educational website were suitable for this purpose. The effectiveness of web-based educational interventions in reducing depression in adolescents has also been shown in other studies^22,23^. The internet-based interventions provide effective and innovative learning experiences and improve adolescent’s access to goals^24^. Nevertheless, contrary to the Lokman et al^25^, improvement of depression in the present study was not stable in the maintenance phase. The initial improvement in depressive symptoms and reversal of symptoms has also reported in other studies ^26,27^. This result might be due to the little duration of the intervention in present study. In addition, there was no specific intervention for environmental contextual factors affecting depression in adolescents^28^. Future studies should use effective strategies to preserve preliminary depression improvements including performing a follow up after the intervention, trying to design web-based messages individual-tailored, and providing other intervention methods in parallel with the web-based intervention, such as teacher training^29^.Significant increase in the mean score of social support in the intervention group immediately after the intervention showed that web-based social support programs may be useful for improving depression in female adolescents. In addition, the formation of a virtual group and online communication with the physician seems to be effective to improve this structure in the present study according to other one^30^.

Self-regulation was enhanced in present study. This finding was consistent with other studies including a reciprocal relationship between depression and self- regulation. Depression can prevent any goal attainment, and a lack of goal setting can lead to depression^31^.

According to this study, the web-based intervention did not influence outcome expectations, which was contrary to the findings of other studies^32^. Nevertheless, the intervention group revealed comparatively high initial levels of outcome expectations and possibly allowed little room for enhancement over time due to a ceiling effect. Moreover, these measures were not sensitive enough to detect cultural complexities related to depression among Iranian female adolescents. For example, some Iranian adolescents did not believe in the positive impact of seeking professional help and stigmatizing them^33^. Considering the importance of this structure in improving depression^34^, future studies should consider how to better address this structure in web-based interventions.

There was no significant difference in self-efficacy between intervention and control groups. Adolescents with low self-efficacy need more guidance for the management of their activities^35^, a long duration of intervention is necessary. This finding was not consistent with other web-based studies^36^. Furthermore, some other factors such as uncertain professional and academic future beliefs, that is common in most Iranian adolescents, due to the social and economic conditions governing the society, can lead to such results^37^.

A lack of correlation between the improvement of the SCT structures and depression changes during the study could be because these structures were not effective factors on depression among Iranian female adolescences or other methods should be used.

In this study, overall satisfaction with the online application component was desirable. Seventy-eight percent of the respondents indicated that they would recommend the website to others with similar psychological conditions. Moreover, majority of female students described the website as “helpful” to “very helpful” to improve depression and "enjoyable" to "very enjoyable" for use. Of course, this finding was views of students who completed the study.

This study was the first to investigate the use of a web-based approach to improve depression based on the SCT constructs. In addition, it developed web site modules based on the findings of a qualitative study applied within 24-weeks.

As limitations, this study was conducted only on females' adolescents that might affect the generalization of findings. Moreover, there was a high rate of attrition, considered in future studies through the increased monetary incentives and the provision of other tangible items ^38,39^. In addition, the website was out of reach after the posttest assessment.

## Conclusion

Improvements in depression, social support, as well as self-regulation in present study were reported. These results, along with self-reported website usage, revealed that probably most helpful programs existing on the website for improving depression were self-regulation and social support applications.

To our knowledge, none other SCT web-based methods were designed for improving depression among students. The results of the present research offer some initial support for effectiveness of a web-based approach for improving depression among female students. Randomized controlled designs are required for the additional assessment of the effectiveness of web-based approaches to improve depression in female adolescents.

## Acknowledgements

The current study was supported by Hamadan University of Medical Sciences. The authors would like to thank all the students and schoolteachers who helped in distributing and collecting the data.

## Conflict of interest

The authors declare that there is no conflict of interests.

## Funding

The current study was supported by Hamadan University of Medical Sciences [grant numbers 9503181264]. The funding body had no role in the study design, the collection, analysis, and interpretation of data, writing the manuscript, or in the decision to submit the manuscript for publication.

## Highlights

- Web-based intervention for the improvement of depression can increase the constructs of the Social Cognitive Theory.
- Increase of the constructs of social cognitive theory with depression had no statistically significant relationships.
- Preservation of the improvement of depression during the post-intervention period requires specific strategies.
- All construct of the Social Cognitive Theory, especially environmental factors, should be considered to reduce depression.
